# Cultured dissociated primary dorsal root ganglion neurons from adult horses enable study of axonal transport

**DOI:** 10.1111/joa.13719

**Published:** 2022-06-21

**Authors:** Robert Adalbert, Stephen Cahalan, Eleanor L. Hopkins, Abdulaziz Almuhanna, Andrea Loreto, Erzsébet Pór, Laura Körmöczy, Justin Perkins, Michael P. Coleman, Richard J. Piercy

**Affiliations:** ^1^ Comparative Neuromuscular Diseases Laboratory, Department of Clinical Science and Services Royal Veterinary College London UK; ^2^ Department of Anatomy, Histology, and Embryology, Faculty of Medicine University of Szeged Szeged Hungary; ^3^ John van Geest Centre for Brain Repair, Department of Clinical Neurosciences University of Cambridge Cambridge UK; ^4^ Andrea Loreto, Department of Physiology, Anatomy and Genetics University of Oxford Oxford UK

**Keywords:** axonal transport, DRG, equine, mitochondria, neurodegenerative diseases

## Abstract

Neurological disorders are prevalent in horses, but their study is challenging due to anatomic constraints and the large body size; very few host‐specific in vitro models have been established to study these types of diseases, particularly from adult donor tissue. Here we report the generation of primary neuronal dorsal root ganglia (DRG) cultures from adult horses: the mixed, dissociated cultures, containing neurons and glial cells, remained viable for at least 90 days. Similar to DRG neurons in vivo, cultured neurons varied in size, and they developed long neurites. The mitochondrial movement was detected in cultured cells and was significantly slower in glial cells compared to DRG‐derived neurons. In addition, mitochondria were more elongated in glial cells than those in neurons. Our culture model will be a useful tool to study the contribution of axonal transport defects to specific neurodegenerative diseases in horses as well as comparative studies aimed at evaluating species‐specific differences in axonal transport and survival.

## Graphical abstract

Here we report the generation of primary neuronal dorsal root ganglia (DRG) cultures from adult horses, containing neurons and glial cells. Cultured neurons varied in size, and they developed long neurites. The mitochondrial movement was detected in cultured cells and was significantly slower in glial cells compared to DRG‐derived neurons. In addition, mitochondria were more elongated in glial cells than those in neurons.

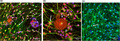

## 1 INTRODUCTION

Small and large animal models are important tools for investigating the pathogenesis of neurodegenerative diseases and developing therapeutic strategies, particularly when diseases are species‐specific. Horses are affected by various neuropathies, including the highly prevalent recurrent laryngeal neuropathy (RLN) of large breeds (Draper & Piercy, 2018), sensory disorders, such as equine trigeminal‐mediated head shaking syndrome (Aleman et al., 2013) and autonomic dysfunction seen in equine grass sickness (Cottrell et al., 1999). In particular, understanding the molecular and cellular basis of disorders that primarily affect long axons, might inform improved understanding of comparable disorders in humans. The recurrent laryngeal nerves (RLn) are the longest nerves in horses (in tall horses, the left RLn can be 2.5 m in length and it is up to 30 cm longer than that of the right RLn) (Draper & Piercy, 2018); in contrast, the longest axons in humans are those that make up the sciatic nerve and its terminal branches, where the nerve can exceed 1 m (Muzio & Cascella, 2022). The study of such diseases in horses is challenging due to the anatomical constraints for work in vivo and to the lack of relevance in vitro experimental models.

Dissociated primary dorsal root ganglia (DRG) cultures from laboratory rodents, usually derived from neonatal or embryonic donors, are widely used to study axon degeneration, regeneration, axonal transport impairment and basic mechanisms of sensory physiology and pain (Baccaglini & Hogan, 1983; Bilsland et al., 2010; Eva et al., 2012; Mellone et al., 2013; Osterloh et al., 2012). Furthermore, DRG cultures are used as models for neurite outgrowth and synapse formation during development and for growth factor‐dependent cell survival (Bayat et al., 2021; De Koninck et al., 1993; Malin et al., 2007; Nowicki et al., 2009; Wong et al., 2015; Wright & Snider, 1995). However, many disorders are unique to larger species, where extended axon length presents a far greater challenge for axonal transport than in rodents, and some have adult onset. There are very few reports of successful primary neuronal culture from larger species and none from horse (Fadda et al., 2016; Gerhauser et al., 2012; Valtcheva et al., 2016).

Mitochondrial dysfunction has been linked to pathogenesis in many neurodegenerative diseases (Lin & Beal, 2006; Wu et al., 2019). Live‐imaging studies of axonal transport in nervous system tissue have largely focused on mitochondria (Mar et al., 2014; Marinkovic et al., 2012; Misgeld et al., 2007). We have previously reported live imaging of mitochondrial transport in peripheral nerve explants and in neuronal cultures from mouse models of neurodegenerative disease and normal aging (Adalbert et al., 2018; Adalbert et al., 2020; Gilley et al., 2012; Milde et al., 2015).

Here, we describe for the first time a protocol suitable for the isolation and culture of primary DRG neurons derived from euthanized adult horses up to 12 years old. Our results show that postnatal horse DRG neurons survive in mixed cell cultures with glial cells for extended periods and establish a neurite network. In addition, we find that mitochondria show different morphologies within DRG neurons and glial cells, and their transport can be assessed in both cell types. This DRG culture method provides a promising model for studying the aetiopathogenesis of specific neurological diseases in horses and for testing therapeutic interventions. Furthermore, the horse primary culture could be used in comparative studies aimed at evaluating species‐specific differences in axonal transport and survival.

## 2 MATERIALS AND METHODS

### 2.1 Animals and isolation of dorsal root ganglia

Tissue samples were collected from eight Thoroughbred horses (age range 3–12 years; 6 geldings and 2 mares) that were subjected to euthanasia as part of separate studies approved by the Home Office (PED82E67D) and local Animal Welfare Ethical Review Board. All horses were determined to be clinically normal and normal on routine neurological examination. Sample collection was achieved within 30 min of euthanasia, performed using an overdose of pentobarbitone administered intravenously.

The cervical vertebrae were bisected, using a band saw and cervical DRGs were isolated from the intervertebral foraminae from within the vertebral canal. A minimum of eight cervical DRGs were pooled from each animal; these were immediately immersed in ice‐cold Hibernate E medium (Gibco). Samples were transported to the laboratory on cold ice. Cervical DRGs from two animals were immersion‐fixed in 10% neutral‐buffered formalin for 48 h and embedded in paraffin. Paraffin‐embedded tissue samples were sectioned at 5 μm thickness, mounted on adhesive glass slides and routinely stained with haematoxylin and eosin (HE) for morphological examination.

### 2.2 Dissociated DRG cultures

**FIGURE 1:**
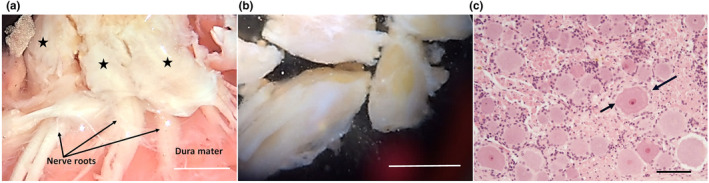
Dissection of horse cervical DRGs prior to dissociation. Extracted ganglia (black stars) are first cleaned of non‐nervous tissue and dura mater (a) and then trimmed of nerve roots before dissociation (b) Scale bars: 1 cm. (c) Histological section of a horse cervical DRG stained with haematoxylin and eosin. The small satellite cells (long arrow) tightly surround the large sensory neurons (short arrow). Scale bar: 100 μm.

Dissociation of DRGs was performed as previously described with slight modifications (Gilley & Coleman, 2010). Surrounding fat, connective tissue, dura mater and nerve roots were carefully removed from each ganglion in a laminar flow hood using sterile instruments (Figure 1a,b). The ganglia were minced into small pieces (approximately 1 ± 2 mm cubes) and dissociated by incubation in 0.025% trypsin (Sigma) in PBS (without CaCl_2_ and MgCl_2_) for 30 min followed by 0.2% collagenase type II (Gibco) in PBS for a further 60 min. Ganglia were then gently triturated using a pipette in Dulbecco's Modified Eagle's Medium (DMEM, Gibco) with 1% penicillin/streptomycin, 50 ng/ml Nerve Growth Factor (NGF)‐2.5S (all Invitrogen) and 10% foetal bovine serum (Sigma). After a 2 h pre‐plating stage to remove some non‐neuronal cells, 5000–10,000 dissociated neurons were plated in a 1 cm^2^ poly‐L‐lysine (20 μg/ml for 1 h; Sigma) and laminin (20 μg/ml for 1 h; Sigma) ‐coated area in the centre of 3.5 cm Ibidi μ‐dishes (Thistle Scientific, Glasgow, UK) using the same medium as above except that 10% foetal bovine serum was replaced with 2% B27 (Gibco). In the first 3 days in culture, 4 μM aphidicolin (Merck) was used to reduce proliferation and viability of non‐neuronal cells. Culture media were replaced every 3 days.

DRG neurons were classified according to soma diameter as ‘small’ (30–49 μm), ‘medium’ (50–69 μm), or ‘large’ (>70 μm). Soma diameter was determined manually on images acquired with a DMi8 inverted microscope (Leica microsystems) using ImageJ software version 1.44 (Rasband, W.S., ImageJ, U. S. National Institutes of Health, Bethesda, MD; http://imagej.nih.gov/ij/, 1997e2012). Soma diameter was measured in 180 neurons, from cultures originating from 8 horses.

### 2.3 Immunocytochemistry

Dissociated cultures were fixed in 4% paraformaldehyde (PFA Sigma Aldrich) in phosphate‐buffered saline (PBS) for 15 min at room temperature (RT). Next, cultures were washed three times in 0.5% PBS, and cells were permeabilized with 0.5% Triton X‐100 in PBS for 10 minutes at RT. To block non‐specific binding, cells were incubated in 5% normal goat serum (Sigma) in PBS for 1 h. Primary antibodies including polyclonal rabbit Tuj 1 (T2200, anti‐βIII tubulin, Sigma, 1:1000), chicken anti‐GFAP (Ab4674, Abcam 1:1000), rabbit anti‐NeuN (Ab104225, Abcam, 1:1000) and rabbit anti‐S100 (Ab34686, Abcam, 1:1000) were applied for 1 h at RT. Then, cultures were washed with PBS 3 times and incubated with Alexa Fluor 488‐conjugated goat anti‐rabbit, Alexa Fluor 568‐conjugated goat anti‐rabbit and Alexa Fluor 647‐conjugated goat anti‐chicken secondary antibodies (Thermo Fisher Scientific; 1:500 in 1% BSA/PBS) for 1 h in the dark. Finally, the cells were washed 3 times with PBS and mounted with Vectashield mounting medium containing DAPI (Vector Labs).

Cell cultures were imaged using a DMi8 inverted fluorescence microscope (Leica microsystems) coupled to a monochrome digital camera (Hammamatsu C4742‐374 95) and a Leica SPE confocal microscope with 63x oil immersion objective and Leica LAS AF software. Image stacks taken were analysed and edited using Fiji software.

### 2.4 Mitochondrial length quantification

Neuronal and glial mitochondria were distinguished based on their localisation and morphology. Mitochondrial length was quantified as described previously (Loreto et al., 2015). DRG cultures were incubated with 20 nM Mitotracker red CMXRos (Invitrogen) for 15 min at 37°C. Fluorescence images were acquired at 37°C on a Zeiss LSM 780 confocal microscope using ZEN 2010 software (Carl Zeiss Inc.) with 100X/1,46 AlphaPlnAPO objective. Mitochondrial length was determined manually by measuring the longest dimension using ZEN (blue edition) software (Carl Zeiss Inc.); specifically, the mean mitochondrial length was calculated from 25–30 mitochondria, measured in five glial and five neuronal cells within one DRG culture originating from each horse (*n* = 8).

### 2.5 Live imaging of mitochondrial transport and image analysis

Mitochondria were labelled with 20 nM Mitotracker red CMXRos (Invitrogen) for 15 min at 37C° and their movement along the neurites was recorded with a Zeiss LSM 780 confocal microscope using a 60x/100x 1.49 NA oil immersion objective (Zeiss). The environment was controlled with a stage top incubator, set at 37°C and 5% CO_2_. Time lapse images of mitochondrial movements were acquired every 1 s for 3 min (180 frames in total). A total of 3–5 movies from different neurons and glial cells were captured from one culture dish for each horse. Individual neurites and glial processes were straightened using the Straighten plugin in ImageJ software version 1.44 (Rasband, W.S., ImageJ, U. S. National Institutes of Health, Bethesda, MD; http://imagej.nih.gov/ij/, 1997e2012). Transport parameters were determined for individual neurites using the Difference Tracker set of ImageJ plugins (Adalbert et al., 2020; Andrews et al., 2010).

## 3 RESULTS

### 3.1 Dissociated DRG neurons from adult horses are viable and develop neurites in cultures

**FIGURE 2:**
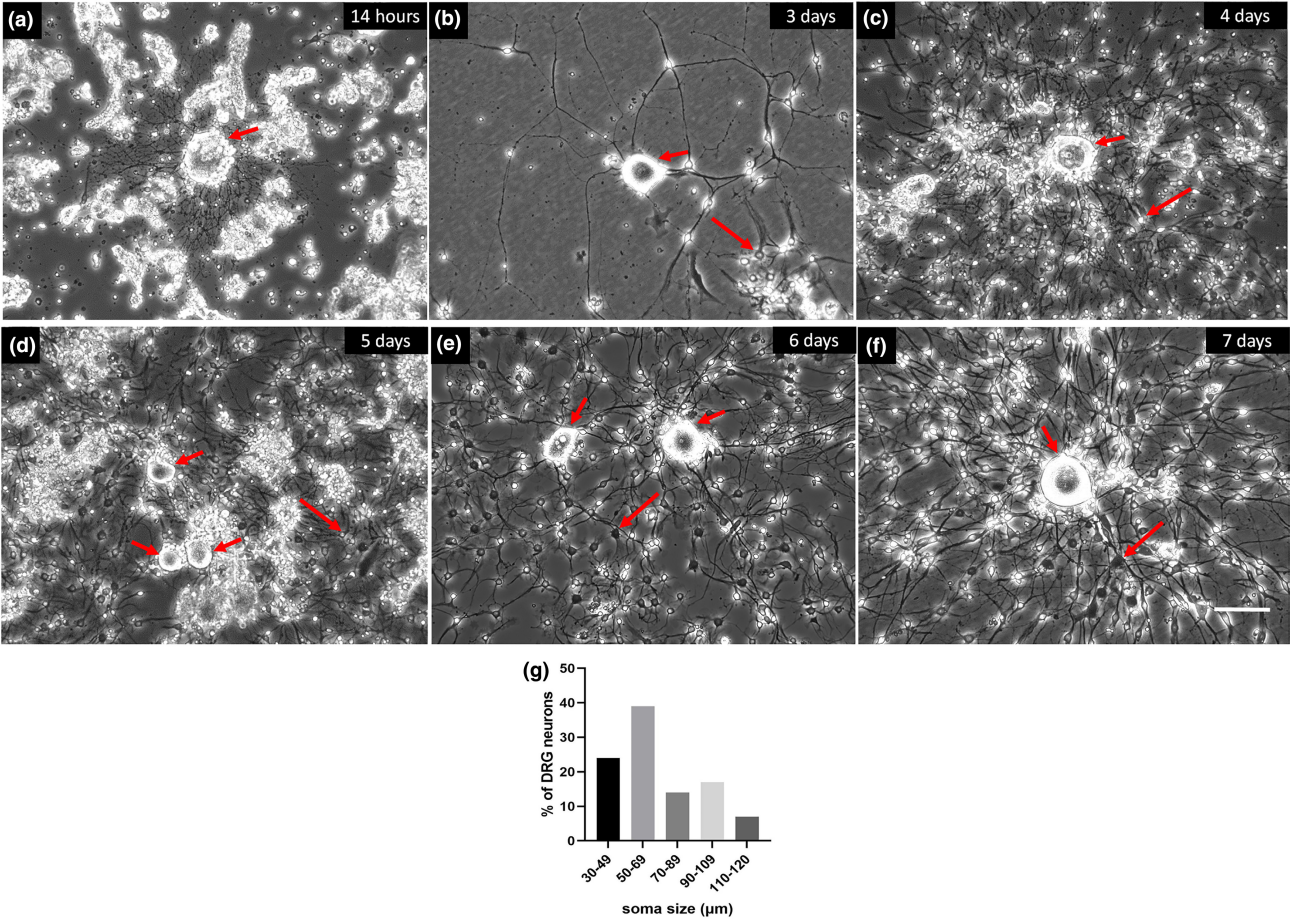
Dissociated horse DRG cultures in vitro. DRG neurons (short arrows) and neurite outgrowth is shown at indicated times in culture (a–f). Neurons are viable and all developed neurites. Glial cells also developed processes in cultures (long arrows). Histogram summarizing the range of neuronal soma diameters in DRG cultures (*n* = 118) (g). Scale bar: 100 μm.

On average, a yield of approximately 5000–10,000 dissociated neurons per DRG was achieved. Similar to DRG neurons in vivo (Figure 1c), cultured dissociated neurons varied substantially in size. Their spherical cell body diameter ranged from 30 μm to 120 μm and showed a centrally located nucleus (Figure 2). Medium and large‐sized neurons were predominant which are likely to represent medium (Aδ) and large (Aβ) neurons.

**FIGURE 3:**
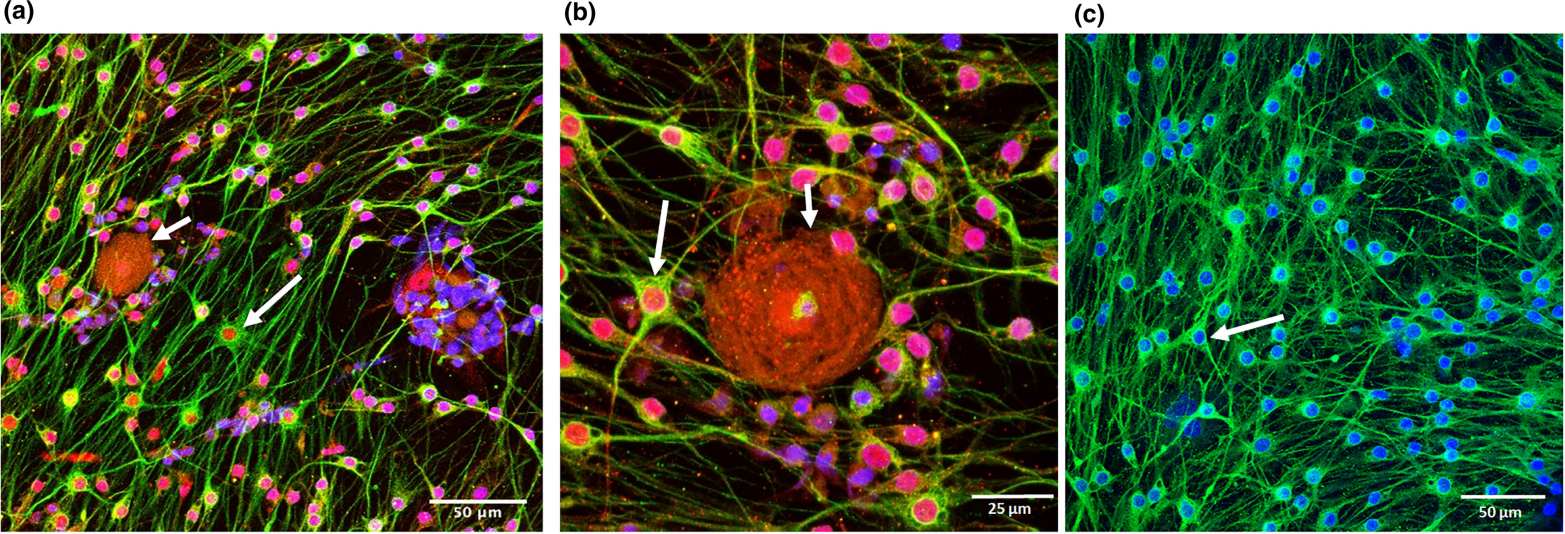
Immunofluorescence characterisation of dissociated DRG culture at 14 days in vitro. Neurons (short arrow) are labelled with anti‐NeuN (red, a, b) and glial cells (long arrows) are stained with GFAP (green, a, b) and S100 (green, c). Glial cells also stained positive for NeuN (a, b). Cell nuclei counterstained with DAPI are shown in blue.

Dissociated neurons were viable for at least 12 weeks in culture and all developed neurites. Neurite outgrowth started within a few hours in culture (Figure 2a) (similar to those of adult mouse DRG neurons in vitro [not shown]). All neurons, regardless of their size, exhibited a high number of neurites (Figure 2a,b) and were NeuN‐positive (Figure 3a,b).

**FIGURE 4:**
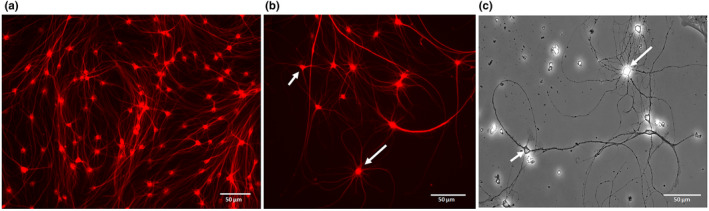
Morphology of glial cells in culture. Immunostaining of the cytoskeletal protein βIII‐tubulin (red) demonstrated extensive growth of the processes and branching at 21 DIV. All the cultures were grown in the same conditions. (a). Larger cells exhibited a higher number of processes compared to the smaller sized glial cells (b and c [phase contrast image]; arrows and short arrows respectively).

Besides DRG neurons, the cultures contained small cells with round nuclei and relatively long processes that expressed GFAP and S100 protein indicating their satellite/schwann cell origin (Figure 3). Intriguingly, the cells also stained positive for NeuN (Figure 3a,b) and βIII tubulin (Figure 4a,b). Their cell body diameter ranged from 5 to 15 μm. Those cells with a larger diameter exhibited a high number of processes compared to smaller cells which had only 2–3 extensions (Figure 4b,c). Process outgrowth was slow, starting with 2 days in culture (Figure 2b) and it reached a plateau at around 21 days (Figure 4).

### 3.2 Mitochondrial morphology varies in culture and their movement can be quantified

**FIGURE 5:**
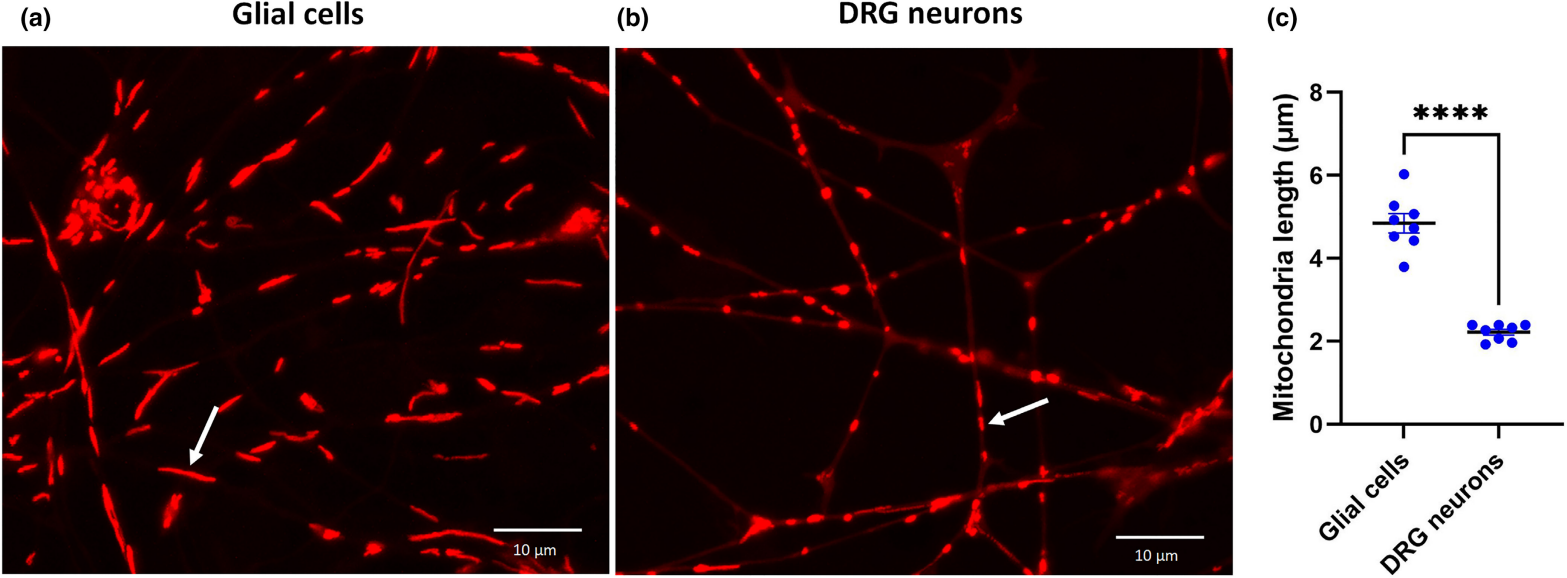
Mitochondrial morphology in horse DRG neuronal cultures 7 DIV. Mitochondria were labelled with Mitotracker red CMXRos (Invitrogen). Glial cell mitochondria (arrow) with an elongated morphology (a) compared to those of neuronal mitochondria (arrow, b); were significantly (****p < 0.0001; paired t‐test) longer than those in neurons (c). Each data point represents the mean value obtained for 25–50 mitochondria measured in each animal. Horizontal bar indicates mean and error bars indicate standard error of the mean. *n* = 8 animals per group.

Neuronal and glial mitochondria were distinguished based on their localisation. Mitochondria labelled with Mitotracker red within DRG neurons appeared different from those within glial cells. In particular, neuronal mitochondria were significantly shorter than those in glial cells at 7 days in vitro (DIV; Figure 5). A similar difference in mitochondrial length between neurons and glial cells was found in older cultures at 14 DIV (not shown).

**FIGURE 6:**
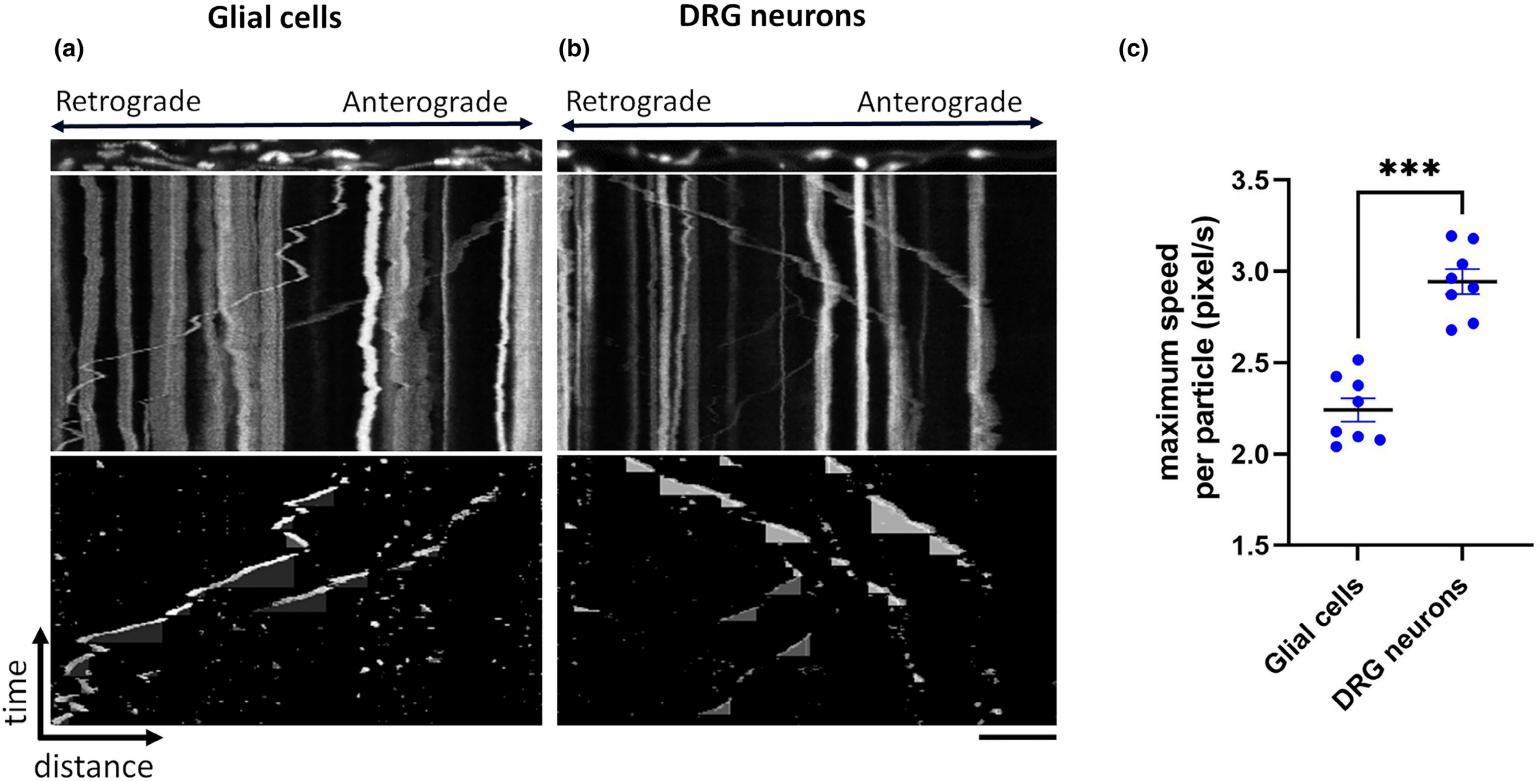
Mitochondrial transport in DRG neurons and glial cells. Representative straightened cellular processes, kymograph and kymograph of tracked particles of mitochondrial transport from glial cells at 14 DIV (a) and neurons at 7 DIV (b). The straightened processes represent the first frame of the time lapse recording (total 180 frames; frame rate 1 fps) that was used to generate the original kymograph. Moving mitochondria were tracked using the ImageJ Difference Tracker (Andrews et al., 2010) set of plugins and another kymograph generated to show successfully tracked particles. Vertical lines indicate stationary mitochondria while lines deflecting to the right or left represent anterograde or retrograde moving mitochondria. Quantification of the maximum speed of mitochondrial movement with cell types indicated (C). Each data point represents the mean value obtained from mitochondria for each animal. Horizontal bar indicates mean and error bars indicate standard error of the mean. Statistically significant difference between groups is indicated (***p < 0.001, paired *t*‐test). *n* = 8 per group. Scale bar: 10 μm.

Mitochondrial movement was present during early growth in the neurites of DRG neurons (Figure 6a) while in the processes of glial cells it was absent during the first 7 DIV and could only be detected in older cultures (typically around 10 DIV) (Figure 6b). In addition, the maximum velocity of mitochondrial movement in glial cells was significantly slower than those in neurons (Figure 6c).

## 4 DISCUSSION

The current study provides a method to produce viable DRG cultures from adult horses that could be used in future for physiological, biochemical and axonal transport studies in horses.

With cell body diameters ranging from 30 to 120 μm, adult horse DRG sensory neurons are large when compared to those of many other species (Delree et al., 1989; Gerhauser et al., 2012; Malin et al., 2007) but similar to those of human and bovine postnatal DRGs in vitro, probably reflecting the very long axons they have to support in vivo (Fadda et al., 2016; Valtcheva et al., 2016). Indeed, a correlation between neuronal soma size and body size has been reported across animal species (Herculano‐Houzel et al., 2014; Ho et al., 1992).

The morphological heterogeneity observed in horse DRG neurons was shown to reflect biochemical and physiological differences between neuronal subpopulations (Russo et al., 2011). The predominance of medium and large‐sized neurons in our cultures is likely to represent a mixed population of nociceptive and non‐nociceptive afferents (Aδ and Aβ ‐fibres) (Crawford & Caterina, 2020) but more work is needed to determine how these findings reproduce those seen in vivo.

Similar to adult DRG cultures from other species, all horse neurons, regardless of their size, exhibited a high number of neurites. The presence of large neurons with numerous neurites is advantageous for morphological studies, and axonal transport and degeneration assays (Adalbert et al., 2020; Fadda et al., 2016; Mellone et al., 2013).

DRG neurons in cultures lose their characteristic (in vivo) pseudo‐unipolar morphology instead becoming multipolar (Takahashi & Ninomiya, 1987). The loss of pseudo unipolarity has been attributed to different factors including the type of growth factor supplementation (Dupraz et al., 2013; Fadda et al., 2016) and activation of transcription factors associated with the regenerative state of dissociated neurons (Frey et al., 2015; Saijilafu Hur et al., 2013).

The small cells in our culture stained positively for GFAP and S100 protein indicating satellite glial and Schwann cell origin (Fadda et al., 2016; Tongtako et al., 2017). Satellite glial and Schwann cells are both derived from neural crest stem cells during embryonic development and share similar gene expression patterns and cellular morphology (George et al., 2018; Hanani, 2005). Recently it was shown that satellite glial cells might represent a population of developmentally arrested Schwann cells (George et al., 2018). Further studies are needed to elucidate which type of the glial cells are predominant in our cultures.

The small, cultured cells were also NeuN and β III tubulin immunopositive. Previously it was shown that glial cells can co‐express GFAP with NeuN and with β III tubulin in cultures (Darlington et al., 2008; Draberova et al., 2008). The reason for the NeuN immunoreactivity of non‐neuronal cells observed in cultures is not fully understood (Gusel'nikova & Korzhevskiy, 2015). It remains to be elucidated whether the co‐expression of β III tubulin along with GFAP signifies bipotential progenitor‐like properties of the horse small cells in cultures or whether it denotes that β III tubulin is a transient constituent of microtubules during gliogenesis. Previously researchers showed that neuronal precursors that give rise to newly generated neurons in DRGs after a crush injury could be represented by satellite glial cells that actively proliferate and are able to differentiate towards the neuronal lineage (Muratori et al., 2015).

The longer mitochondrial morphology within the glial cells could be attributed to their increased fusion in these cell types due to their metabolism or differentiation stage (Jackson & Robinson, 2018; Seo et al., 2018). Mitochondrial oxidative capacity is thought to vary inversely with size (Bertoni‐Freddari et al., 2003; Jackson & Robinson, 2018). As shown in mice, (Jackson & Robinson, 2018; Rinholm et al., 2016), mitochondrial movement was slower in cultured equine glial cells than in neurons. The rates of mitochondrial movement in astrocytes match the rates of mitochondrial movement along actin microfilaments (Morris & Hollenbeck, 1995). Therefore, we speculate that the lower velocity of mitochondrial movement observed in glial cells in our equine cultures might be explained by expression of different motor or adaptor proteins involved in transport in these cells compared to neurons.

Our mixed cell culture recapitulates the organisational architecture between neurons, neurites and glial cells in the peripheral nervous system. It will be important now to use our culture model to study axonal transport defects that might underlie certain neurodegenerative diseases in horses and to test therapeutic interventions directly. Furthermore, the horse primary culture could be used in dynamic growth assays, studies on neurite regeneration, ageing and pain involving the peripheral nervous system as well as comparative studies aimed at evaluating species‐specific differences in axonal transport and survival.

## AUTHORS’ CONTRIBUTIONS

R.A., S.C., J.P., M.P.C. and R.J.P. carried out research design. R.A., S.C., E.L.H., A.A., A.L. and J.P. carried out experimental work. R.A., E.P., A.L., L.K., M.P.C. and R.J.P. contributed to data analyses and interpretation. R.A. and R.J.P. contributed to writing the manuscript.

## FUNDING INFORMATION

This study was funded by the UK Horserace Betting Levy Board.

## Data Availability

The data that support the findings of this study are available from the corresponding authors upon reasonable request.
